# Medical Accident Investigation System of Japan in Malpractice Litigation: A Non-Punitive Reporting System?

**DOI:** 10.31662/jmaj.2025-0232

**Published:** 2025-11-21

**Authors:** Masayuki Ohira, Satoru Makita, Masaki Takao

**Affiliations:** 1 Department of General Internal Medicine and Clinical Laboratory, National Center of Neurology and Psychiatry National Center Hospital, Kodaira, Tokyo, Japan; 2 Jinho Law Office, Minato, Tokyo, Japan; 3 Makita Law Office, Shinjyuku, Tokyo, Japan

**Keywords:** malpractice lawsuits, medical accident investigation system, patient safety, non-punitive reporting

## Abstract

**Introduction:**

The investigation and reporting of medical accidents are important to prevent medical errors and ensure patient safety. A successful reporting system should be non-punitive, meaning that reporters are free from any reprisals that arise owing to their reporting of a medical incident.

**Methods:**

In this study, we reviewed all lawsuits involving reports in Japan’s Medical Accident Investigation System, identified using a Japanese database of lawsuits (https://www.westlawjapan.com/). We examined the basic characteristics of medical accident cases and how these reports were cited in judgments in medical malpractice litigation.

**Results:**

A total of 13 cases, including 15 reports, were collected in this study. Two judgments cited two kinds of reports: those conducted in-hospital and reports prepared by the Medical Accident Investigation and Support Center. Twelve reports (80.0%) were provided by the bereaved family, and four reports (26.7%) were entered into evidence as exhibits for the determination of negligence. Nine reports (60.0%) were cited as evidence of negligence; among these, six reports included content establishing negligence among medical professionals. At least three reports provided grounds for the establishment of negligence in the judgment.

**Conclusions:**

We described the basic characteristics of malpractice lawsuits related to the medical accident reporting system established under Japanese law. The current situation of reports provided in medical malpractice lawsuits in Japan indicates discrepancies between their use and the purpose of a non-punitive reporting system. Systems to ensure patient safety should be separate and distinct from those intended to report negligence by medical professionals.

## Introduction

Prevention of medical error is crucial for patient safety. For this purpose, a system for the investigation and reporting of medical accidents or incidents is also important. Prosecuting and punishing individual errors without a review of the system has been criticized for violating the principles of medical safety and is recognized as an obstacle to improving patient safety ^(1)^. In 2005, the World Health Organization (WHO) issued recommendations on patient safety, known as the WHO Draft Guidelines (hereinafter, the guidelines) ^(2)^. These guidelines aim to ensure patient protection by establishing a system for reporting and learning from medical accidents, including those resulting in treatment-related deaths. The guidelines highlight seven key characteristics necessary for a successful reporting system. One characteristic is that of being “non-punitive,” meaning that reporters are free from fear of reprisals that arise as a result of reporting an incident ^(3)^.

Japan’s new Medical Accident Investigation System (MAIS) was established in October 2015 under the Medical Care Act (hereinafter, the Act) as a system for the reporting and investigation of medical incidents. The MAIS involves in-hospital committees and third-party experts (the Medical Accident Investigation and Support Center, as well as support organizations) in the investigation and prevention of medical accidents ^(4), (5)^. When a medical accident or incident occurs and is reported, the medical facility involved is expected to conduct an investigation and submit a report to the Medical Accident Investigation and Support Center. In the Act, a “medical accident” is defined as a death or stillbirth that was allegedly the result of care provided by one or more medical professionals and that was unforeseen by the administrator. When the administrator judges that there has been a medical accident as defined in the Act, the hospital conducts an in-hospital investigation, collecting and analyzing data to investigate the causes of the incident. During this procedure, a report or similar document is generated that includes results of the in-hospital investigation (hereinafter, in-hospital report), which is provided to the bereaved family in some cases ^(6)^. Additionally, the Medical Accident Investigation and Support Center may investigate the reported case at the request of the hospital or the family concerned. The report generated as the result of such an investigation (hereinafter, center report) is provided to both the hospital and the bereaved family ^(5)^.

The guidelines state that ideally, reporting systems should be designed for learning purposes and that those systems should adhere to principles such as non-punitiveness, confidentiality, and independence ^(3)^. The Ministry of Health, Labour and Welfare of Japan states that the MAIS is consistent with the above principles and aligns with learning-oriented systems, as outlined in the guidelines ^(7)^. The MAIS is said to be aimed not at pursuing accountability among medical professionals for past incidents but at preventing the recurrence of medical accidents to ensure patient safety in future medical care ^(8)^. However, the use of MAIS reports in medical malpractice litigation is not restricted in Japan ^(9)^.

To our knowledge, no previous studies have analyzed medical malpractice litigation citing in-hospital reports or center reports as documentary evidence. If these reports are used as evidence in medical malpractice litigation, this could lead to civil or criminal punishment and contradict the critical characteristic of the MAIS as being a non-punitive system. Therefore, in this study, we aimed to investigate whether reports generated in the MAIS have been used as documentary evidence in medical malpractice judgments, and if so, for what purpose; in other words, we sought to identify whether reports were cited for the purpose of fact-finding or for determining negligence. Through this analysis, we aimed to clarify whether the MAIS is being used for its original purpose, namely, achieving patient safety.

## Materials and Methods

### Study design, data, and settings

We conducted a retrospective review of medical malpractice cases related to the MAIS. Cases were identified in a search of the Westlaw Japan nationwide online database, which includes data on legal cases in the country (https://www.westlawjapan.com/). Westlaw is a comprehensive legal research engine that has been used in various types of medical malpractice research in other countries ^(10), (11), (12)^. Westlaw Japan, the Japanese version of Westlaw, represents the largest legal case database in Japan, covering more than 300,000 cases dating back to the prewar era ^(13)^. On February 16, 2025, we conducted a basic search to identify medical malpractice cases using only one keyword, “medical accident investigation,” or *Iryogikochosa* in Japanese.

In the first stage of the present research, the date of each judgment was checked to exclude cases prior to October 1, 2015, when the law outlining the MAIS was enacted in Japan. In the second stage of research, the content of all judgments was screened to confirm that MAIS reports had been cited in each identified case. We also collected the basic characteristics of all cases, including the docket number, name of the court, characteristics of the plaintiff and defendant, the patient’s disease or condition, and the final judgment (i.e., dismissal or acceptance).

In the third research stage, among judgments citing MAIS reports as evidence, we analyzed how each report was entered into litigation. Specifically, we recorded the parties who provided these reports as evidence and the categorization of each report according to the type of evidence. To confirm this, we used the exhibit number assigned to any MAIS reports appearing in a case judgment. The exhibit number for evidence in medical malpractice lawsuits in Japan usually contains information regarding the party submitting the evidence, the bereaved family or medical professional (plaintiff and defendant, respectively), and a classification as type A, B, or C ^(14)^. Documents classified as type-A exhibits comprise documentary evidence related to facts that detail the course of medical treatment, nursing care, medication, and other clinical procedures. Documents classified as type-B evidence may be used in the determination of negligence and include records such as evaluations of relevant medical procedures, general medical knowledge, and other similar information. Type-C documents encompass any documents used in support of monetary damages to be awarded, as well as any other documentary evidence not categorized as type A or B.

In the fourth and final research stage, we scrutinized the way in which the court cited MAIS reports in the judgment. We classified cited reports according to the purpose of their citation: fact-finding, determination of negligence, or other purposes such as determining causation or evaluating monetary damages. If the court cited a MAIS report as evidence for the determination of negligence, we also assessed whether the content of the cited report supported or opposed a claim of negligence and whether the court made a determination of negligence in related issues.

All of these stages in the present research were implemented by one researcher (MO), who is a medical doctor certified as a Fellow of the Japanese Society of Internal Medicine, as well as a licensed attorney in Japan and a member of a law firm in Tokyo. The third and fourth stages were independently conducted by another researcher (SM), a licensed Japanese attorney and member of a law firm in Tokyo.

### Statistical analysis

Continuous variables are expressed as median (interquartile range, 25%-75%), and categorical variables are presented as frequency and percentage. The degree of agreement between MO and SM was quantified according to absolute agreement (AG) and Cohen’s kappa (κ). AG refers to the probability that two researchers rate the same items in the same category in the same way. Cohen’s κ quantifies the agreement exceeding that owing to chance, such that a value of zero indicates agreement by chance. This categorization of chance-corrected agreement was proposed by Landis and Koch: a value of <0.2 is considered poor agreement, 0.21-0.4 fair, 0.41-0.6 moderate, 0.61-0.8 strong, and >0.80 near-complete agreement ^(15)^. These measures of interrater agreement were calculated using EZR (Saitama Medical Center, Jichi Medical University, Saitama, Japan), a graphical user interface for R (The R Foundation for Statistical Computing, Vienna, Austria) ^(16)^.

## Results

**Figure 1.**
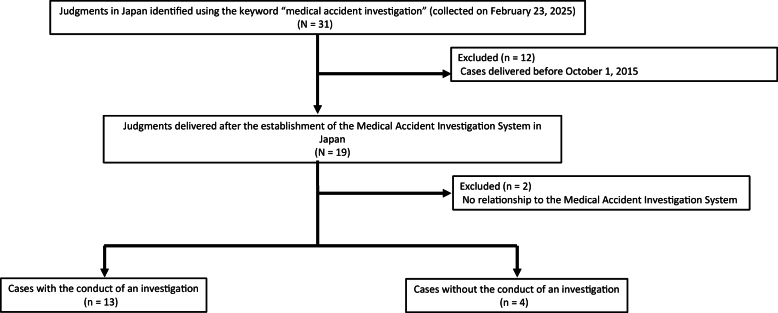
Study flowchart. The flowchart illustrates the selection of 31 medical malpractice litigation cases; 14 cases were excluded because they were not relevant to the Medical Accident Investigation System (MAIS) or were rendered before October 1, 2015. Four were also excluded because no investigation was conducted.

First, we identified 31 cases of medical malpractice litigation using only the keyword “medical accident investigation,” or *Iryogikochosa* in Japanese. All identified cases were civil cases; no criminal cases were found in the database search. Among the 31 cases, 12 were excluded because they were rendered before October 1, 2015. Two cases were also excluded because they were not related to the MAIS in any context. Among the 17 remaining cases, we confirmed that an actual investigation was conducted in 13 cases, from the context of the judgment. In the other four cases, no investigation appeared to have taken place (Figure 1).

The basic characteristics of the 13 investigated cases above are summarized in Table 1. The 13 cases involved nine male patients and four female patients. All patients except one (case 7) had died. The average patient age was 46.4 years (range, 29-71.5 years). In addition to medical institutions, medical staff such as doctors were among the defendants in three cases (cases 3, 6, and 7). The specialty of the medical professionals involved was psychiatry in three cases, obstetrics in three cases, and surgery in two cases. The final judgment was in favor of the plaintiff in six cases and in favor of the defendant in seven cases.

**Table 1.** Cases Where a Medical Accident Investigation Was Conducted and Its Report Was Cited. Summary of demographic and clinical details of 13 medical malpractice cases where investigations were conducted.

Case	Judgment date	District Court	Case number	Patient’s disease or condition	Defendant	Physicians’ specialty	Conclusion of the judgment
1	January 31, 2020	Kanazawa	2018(Wa)410	Schizophrenia and acute pulmonary thromboembolism	Hospital	Psychiatry	Dismissed
2	March 30, 2020	Kanazawa	2016(Wa)539	Hepatocellular carcinoma and hemorrhagic shock after diaphragmatic injury	University hospital	Internal medicine	Accepted
3	February 17, 2021	Kyoto	2017(Wa)2052	Delivery with paroxysmal nocturnal hemoglobinuria	University hospital The President Doctors	Hematology Obstetrics	Accepted
4	August 27, 2021	Tokyo	2020(Wa)27978	Hemorrhagic shock following endoscopic submucosal dissection	Hospital	Surgery	Accepted
5	September 30, 2021	Tokyo	2019(Wa)8007	Death following blood transfusion	Hospital	Unclear	Dismissed
6	November 30, 2021	Yokohama, Kawasaki branch	2016(Wa)894; 2017(Wa)316	Hemorrhagic shock after vertebral artery injury following internal jugular vein central venous catheterization	Hospital Doctor	General medicine	Dismissed
7	January 28, 2022	Osaka	2017(Wa)9561	Severe birth asphyxia and hypoxic-ischemic encephalopathy	Hospital Doctor	Obstetrics	Dismissed
8	February 3, 2022	Tokyo	2021(Wa)21729	Anasarca with unknown cause	Hospital	Surgery	Dismissed
9	March 23, 2023	Tokyo	2018(Wa)31952	Surgery for right mandibular fracture	Hospital	Anesthesiology	Dismissed
10	April 26, 2023	Kyoto	2022(Wa)1378	Depression	University hospital	Psychiatry	Accepted
11	August 4, 2023	Kobe	2019(Wa) 1604	Schizophrenia and neuroleptic malignant syndrome	Hospital	Psychiatry	Accepted
12	January 31, 2024	Yokohama	2019(Wa)1363	Amniotic fluid embolism	Clinic	Obstetrics and gynecology	Dismissed
13	April 26, 2024	Kobe	2021(Wa)703	Hypopharyngeal carcinoma, esophageal carcinoma, and iatrogenic pneumothorax	Hospital	Unclear	Accepted

We summarize the features of cited MAIS reports in Table 2. In cases 3 and 6, two final MAIS reports that were generated after both an in-hospital and a center investigation were cited as evidence. In case 7, the MAIS report itself was not cited in the final judgment; only a document containing the response to the bereaved family based on the results of the in-hospital investigation was used as evidence. We included this type of document as well as MAIS reports in this study. Therefore, a total of 15 reports were found to be cited as documentary evidence of medical malpractice. Most reports (80.0%) were provided by the bereaved family. Approximately one-fourth of reports (26.7%) were classified as type-B evidence, meaning that they were pertinent for the determination of negligence. In case 10, the MAIS report was not classified as any type. In this third stage of the research, the interrater reliability was good (AG 100%, Cohen’s κ = 1.00, 95% confidence interval [CI]: 1.00-1.00).

**Table 2.** Characteristics of Medical Investigation Reports Used as Evidence for Medical Malpractice. Overview of characteristics of 15 reports cited as evidence in 13 malpractice cases, including both in-hospital reports and center reports.

Characteristics	N = 15	
Preparer of the report		
Hospital	13	(86.7)
Center	2	(13.3)
The party that introduced a report in litigation		
Patient: n (%)	12	(80.0)
Hospital: n (%)	3	(20.0)
Classification of reports according to the type of evidence		
Type A: n (%)	7	(46.2)
Type B: n (%)	4	(26.7)
Type C: n (%)	3	(20.0)
Unclassified: n (%)	1	(6.7)
Use of the report as evidence in litigation		
Fact-finding	10	(66.7)
Determination of negligence	8	(53.3)
Other	2	(13.3)

Nine reports (60.0%)―seven in-hospital reports, and two center reports―were cited as evidence of negligence (AG: 93.3% agreement, κ = 0.86, 95% CI: 0.59-1.12). Both in-hospital and center reports were cited in two judgments (cases 3 and 6); therefore, a total of seven cases (cases 1, 3, 4, 6, 7, 9, and 10) cited these reports as evidence of negligence. Among these nine reports, six included content establishing negligence by a medical professional (AG: 88.9% agreement, κ = 0.78, 95% CI: 0.34-1.20). With citation of these reports, four reports were related to a court decision of negligence and five were not (AG: 100% agreement, κ = 1.00, 95% CI: 1.00-1.00).

In three judgments (cases 1, 4, and 10), the content of the reports was deemed to support negligence by a medical professional, and the courts involved established negligence, citing issues in these reports. In case 1, the patient was hospitalized in a psychiatric institution, where physical restraint was applied; the patient later died due to pulmonary thromboembolism. The in-hospital report pointed out that patients subjected to physical restraint must wear elastic stockings (compression stockings) under the direction of a physician. Citing this part of the report as one piece of evidence, the court declared that the hospital had a duty of care to ensure that the patient wore elastic stockings during the period of physical restraint but failed to do so. In case 4, the patient underwent endoscopic submucosal dissection (ESD) at the hospital and subsequently died due to hemorrhagic shock. The in-hospital report concluded that the patient had no indication for ESD and that erroneous decisions made by the physician during ESD led to hemorrhagic shock and the patient’s death. In addition to this content in the report, the court pointed out that the defendants (i.e., the medical professionals) did not actively refute the report. The court determined that the physicians breached their duty to provide appropriate medical care, concluding that their negligence stemmed from implementing ESD for a patient who did not have an indication for this procedure. In case 10, the patient, who was hospitalized in the psychiatric department of a university hospital, left the hospital without authorization and subsequently died by suicide. The in-hospital report stated that psychiatric patients who leave the hospital without permission should be regarded as high risk and that their information needs to be appropriately shared by medical professionals caring for such patients. The court established negligence by the medical professionals involved, stating that, when accompanying the patient outside the closed psychiatric ward, physicians had a duty to remain by the patient’s side and ensure that they could monitor the patient’s behavior, even when the patient was in the restroom.

The degree of agreement between researchers (MO and MS), i.e., AG and Cohen’s κ, was nearly complete throughout the third and fourth stages in this study.

## Discussion

In this study, we described the introduction and use of MAIS reports as evidence in Japanese medical malpractice litigation. To our knowledge, no previous study has examined past lawsuits in Japan from this viewpoint. Additionally, we found that some reports were cited in determining negligence on the part of medical professionals. A few reports were actually instrumental to the court in establishing negligence in its judgment. These findings may directly contradict the original purpose of the MAIS, namely, to prevent future medical accidents and improve patient safety. Owing to substantial differences in legal systems across different countries, a simple comparison of medical error reporting systems is difficult and inappropriate. However, reluctance on the part of health care professionals to report medical errors seems to be common. For instance, in the United States, federal law―specifically, the Patient Safety and Quality Improvement Act―provides protections against discovery in litigation for patient safety work products ^(17)^. Nevertheless, state laws govern state courts and may vary state by state, resulting in this protection failing to be uniformly applied across all states ^(18)^.

The features of MAIS reports in medical malpractice litigation showed that patients or bereaved families actively used these reports as evidence in their claims against medical professionals. Nearly all reports were provided to the court by the bereaved family. Half of the MAIS reports in this study were categorized as type-A exhibits, meaning that they were intended to be used for fact-finding, i.e., establishing the course of medical treatment, nursing care, medication, and other clinical procedures ^(14)^. However, more than half of the reports were cited in the determination of negligence in the judgment. This discrepancy exists because the categorization of exhibits for evidence does not prohibit the court from using such documents for other purposes. According to Japan’s Ministry of Health, Labour and Welfare, the law has never prohibited the possible use of MAIS reports as evidence in litigation ^(9)^. We cannot determine whether the use of reports from the MAIS described in this study is in line with the WHO Draft Guidelines, i.e., being learning-oriented, non-punitive, confidential, and independent. This situation may lead medical professionals to avoid using the MAIS because reports generated via this system may result in a determination of medical negligence in future litigation. To achieve the original goal of medical incident reports, legal measures prohibiting their use in court are necessary. Ideally, they should be accompanied by systemic support that addresses the inequity of access to evidence between parties in medical malpractice litigation, as discussed below.

The determination of negligence regarding issues cited using MAIS reports was not clearly related to the content of these reports. This could indicate that some courts did not establish medical negligence purely on the basis of the reports. The purpose of the MAIS is to ensure patient safety in future medical care by preventing the recurrence of medical accidents; its purpose does not include pursuing accountability for incidents that have already occurred ^(9)^. The reports generated within the system should align with the objectives of the MAIS. Therefore, it is reasonable that courts should determine negligence by medical practitioners using evidence that is separate and distinct from the content of MAIS reports.

The reason why MAIS reports were provided to the court as evidence, mostly by the bereaved families, remains unclear. The most likely reason arises from the Japanese judicial system in civil procedures. When a medical malpractice lawsuit is filed with a court, the plaintiff has a responsibility to show that the patient was given substandard care ^(19)^. In general, the plaintiff has little expert knowledge or documentary evidence related to medical care. To account for this disadvantage in litigation and to provide evidence that is sufficient to convince a judge that medical professionals involved in the case were negligent, MAIS reports could serve as a very important and useful tool for patients or bereaved family members. This problem within the Japanese judicial system may lead to the misuse of MAIS reports.

A few MAIS reports likely directly affected the establishment of negligence or medical malpractice in this study. We found that some MAIS reports were used effectively to assign civil liability to medical professionals. This meant that the reporting of a medical accident or incident led directly to the reporters experiencing repercussions in civil litigation procedures; this outcome can adversely affect patient safety, resulting in medical professionals being reluctant to share information so as to learn from past medical incidents. It is essential to consider the establishment of a distinct system that addresses evidentiary inequities between parties in medical malpractice litigation and ensures that the MAIS is used exclusively for its original purpose, namely, to improve patient safety.

In this study, we collected information regarding simple final decisions (i.e., acceptance or dismissal) in each case; information regarding monetary damages was not collected. Some past studies on medical malpractice lawsuits in Japan have included the value of monetary damages awarded ^(20)^. However, this has limited meaning in assessing malpractice litigation in Japan from a scientific viewpoint. Damages and payments in medical malpractice litigation in Japan closely follow those for personal injuries resulting from accidents, such as automobile accidents. These damages are determined with reference to what is colloquially known as the “Red Book,” a guide for calculating damages following traffic accidents that is published annually by a lawyers’ association in Tokyo ^(21)^. Therefore, damages in malpractice litigation in Japan are calculated based on fixed factors such as the age, sex, and wage-earning status of those involved, as well as the costs of hospitalization, rather than the degree of negligence. When examining medical malpractice litigation scientifically, the value of monetary damages awarded in judgments is largely unimportant.

### Limitations

This study has several limitations. First, the number of included cases was small. Cases in this study were identified using a database (https://www.westlawjapan.com/). Additionally, information on the settlement of cases is unavailable to most researchers in Japan. Statistics on medical malpractice litigation suggest that approximately half of cases are settled by reconciliation, and their results are therefore unknown to the public ^(22)^. This could lead to selection bias because it is conceivable that medical professionals are more inclined to settle medical malpractice lawsuits, especially when MAIS reports contain information that is unfavorable to them. Second, we cannot rule out the possibility of missed cases that should have been included in the study owing to the systemic barriers involved with respect to Japanese court judgments. The court is not required to clearly describe every piece of documentary evidence within the content of the judgment ^(23)^. If the court cited a report using only the exhibit number, it was impossible to identify those cases that were potentially eligible for inclusion in this study.

### Conclusions and implications

In this study, we investigated MAIS reports cited as documentary evidence in Japanese medical malpractice litigation. In some cases, these reports were used to support decisions regarding negligence by medical professionals. Such uses may contradict the basic idea of the MAIS and diminish the value of the medical investigation system itself. Medical professionals should be aware of how MAIS reports can be used in the future to ensure that the MAIS remains an appropriate incident reporting system aimed at facilitating patient safety.

## Article Information

### Acknowledgment

We thank Analisa Avila, MPH, ELS, of (https://jp.edanz.com/ac) for editing a draft of this manuscript.

### Author contributions

Masayuki Ohira contributed to the study concept and design, implementation of the study, analysis of the results, and writing of the manuscript. Satoshi Makita contributed to implementation of the study. Masaki Takao contributed to critical revision of the manuscript, funding acquisition, administrative support, and supervision. All authors have agreed to the final submitted manuscript.

### Conflicts of Interest

None

### IRB approval

This study focused purely on publicized judgments and did not include any patient information. Therefore, IRB approval was not needed.

### ORCID iD

Masayuki Ohira: 0000-0002-2754-7212

Masaki Takao: 0000-0002-5392-996X
